# Impacts of Climate Change on the Timing of the Production Season of Maple Syrup in Eastern Canada

**DOI:** 10.1371/journal.pone.0144844

**Published:** 2015-12-18

**Authors:** Daniel Houle, Alain Paquette, Benoît Côté, Travis Logan, Hugues Power, Isabelle Charron, Louis Duchesne

**Affiliations:** 1 Direction de la recherche forestière, Ministère des Forêts de la Faune et des Parcs du Québec, 2700 Einstein, Québec, QC G1P 3W8, Canada; 2 Consortium sur la climatologie régionale et l’adaptation aux changements climatiques (Ouranos), 550 Sherbrooke W, Montréal, QC H3A 1B9, Canada; 3 Centre for forest research, Université du Québec à Montréal, P.O. Box 8888, Centre-ville Station, Montréal, QC H3C 3P8 Canada; 4 Dep. of Natural Resource Sciences, McGill University, 21,111 Lakeshore Rd., Ste. Anne de Bellevue, QC H9X 3V9 Canada; University of Guelph, Canada, CANADA

## Abstract

Maple syrup production is an important economic activity in north-eastern North-America. The beginning and length of the production season is linked to daily variation in temperature. There are increasing concerns about the potential impact of climatic change on this industry. Here, we used weekly data of syrup yield for the 1999–2011 period from 121 maple stands in 11 regions of Québec (Canada) to predict how the period of production may be impacted by climate warming. The date at which the production begins is highly variable between years with an average range of 36 days among the regions. However, the average start date for a given region, which ranged from Julian day 65 to 83, was highly predictable (r^**2**^ = 0.88) using the average temperature from January to April (T_J-A_). A logistic model predicting the weekly presence or absence of production was also developed. Using the inputs of 77 future climate scenarios issued from global models, projections of future production timing were made based on average T_J-A_ and on the logistic model. The projections of both approaches were in very good agreement and suggest that the sap season will be displaced to occur 15–19 days earlier on average in the 2080–2100 period. The data also show that the displacement in time will not be accompanied by a greater between years variability in the beginning of the season. However, in the southern part of Québec, very short periods of syrup production due to unfavourable conditions in the spring will occur more frequently in the future although their absolute frequencies will remain low.

## Introduction

Maple syrup production from sap goes back to the first nation peoples of north-eastern North-America [1]. With time, the methods were modernized and maple syrup production is now an important economic activity for the region. Sugar maple (*Acer saccharum* Marsh.), a dominant species throughout north-eastern North-America, is the most-used species because of its high sugar sap content (2–3%) [2]. In addition to sap sweetness, there is increasing interest in the chemical composition of maple syrup, particularly the phenolic compounds, because of their antioxidant, antiradical and antimutagenic properties [3].

The period of syrup production [4] as well as the annual yield [5, 6] have been shown to depend on climatic conditions, and therefore vary between years and regions. Recently, sugar maple producers in the province of Québec (which is responsible for 75% of the world production) have expressed concerns about the high year to year variability in the timing of the season (Houle, unpublished data). Similarly, in a survey conducted among producers of Québec, Ontario, Atlantic Canada and northeastern USA, 73% mentioned that start of the season was showing increasing between years variability [7].

Sap volume and sugar content are associated to climatic conditions that prevail during the production season, as well as in the months preceding it [8–11]. Several processes are involved in initiating spring sap production, which is based on alternating positive and negative pressure in the trunk and branches which is favoured by freezing temperatures at night followed by above-zero temperatures during the day (freeze/thaw cycle) [8, 12, 13].

The physiological processes involved, as much for volume as for sugar content, can be related to a few determinant conditions. Some authors noted that the ideal climatic conditions for sap production are temperatures close to 0°C at night rising above 4°C during the day [11, 12, 14]. Others identified the maximum daily temperature [8] or the difference between the minima and maxima as factor determining production [13]. Climatic conditions in the months preceding sap production can also be determinant [6]. Precipitation (snow or rain) between October and April (inclusively) are positively correlated to syrup production in the following spring [11]. Cold winters are also positively correlated to yield because they are associated with increased sugar content in sap [6]. Warm temperatures in April may abruptly halt production by favouring bud break and the proliferation of microorganisms in the tap [6].

Because of the close links between climate and maple syrup production, climatic change is likely to have a strong impact on the industry [4, 6]. Predicted climate change effects for north eastern North-America include an increase in annual temperature and precipitation which will be particularly strong during winter, as well as a reduction in the duration of the snow cover [15]. Below freezing winter temperatures must last long enough for the tree to begin the dehardening process necessary for the beginning of sap production [16]; should this period become too short, tree physiology and vigor could be affected which would reduce sap production. Although no significant trends are projected in the number of freeze/thaw cycles [15], the period of the year where the frequency of those cycles is at its peak is expected to happen sooner in the season. The possible impacts of these changes on maple syrup production are many. Because of its dependence on freeze/thaw cycles, the production season should occur earlier in time [4]. Duchesne et al. [6]suggested that the period of sugar maple production will have to be shifted 12 and 19 days earlier in 2050 and 2100 respectively, in order to capture the optimum climatic conditions necessary to maintain the syrup production at its current level.

This paper aims at investigating the impact of climate change on the timing and duration of the sap season. To date, the temporal variability of syrup yield has been studied mainly on an annual basis [1, 6], or on finer temporal scales (days) but for only one specific site at a time for a one-year period [11, 13]. As a result, models capable of taking into account the variability in fine temporal scales and regional differences in timing of the sap season are still lacking. Here, we use a weekly data set of maple syrup production from 121 sugar maple producers located in 11 regions of Québec for a period of 13 years along with climate data to build models of maple syrup production timing. These models are then used to make projections of the impact of climate change on the timing (beginning and end) of maple syrup production based on 77 climate change scenarios issued from global climate models. We hypothesize that the period of syrup production will occur sooner in spring and that it will become more variable between years.

## Methods

### Maple syrup production and climatic data

**Fig 1:**
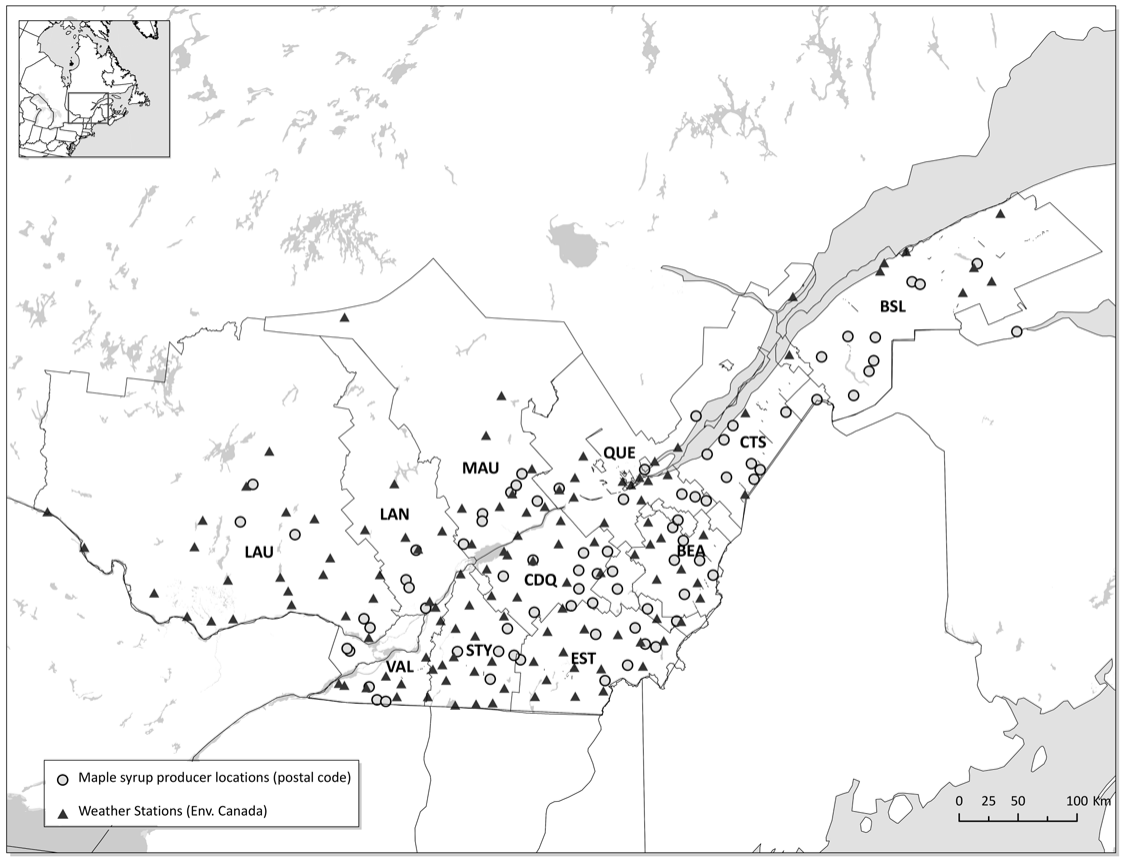
Study area showing the 11 regions, the location of the producers (grey dot) participating in the survey (1999–2011) and the weather stations (black triangle). BSL: Bas-Saint-Laurent; CTS: Côte-du-Sud; BEA: Beauce; QUE: Québec; MAU: Mauricie; LAN: Lanaudière; EST: Estrie; LAU: Laurentides, Outaouais, Abitibi-Témiscamingue; CDQ: Centre-du-Québec; STY: Saint-Hyacinthe; VAL: Valleyfield.

Weekly maple syrup production data (kg per tap) for the 1999–2011 period were obtained from surveys collected by the « *Fédération des Producteurs Acéricoles du Québec* » (FPAQ) from 121 producers scattered over 11 regions in Québec (Fig 1 and Table 1). Weekly data from all contributing producers from a given region were aggregated to obtain a total of 143 observations (13 years times 11 regions) for the beginning, end and length of the sap production period. Daily climate data from several weather stations in each region (Fig 1) were used to compute the weekly climate variables needed for the predictive model. The modeling was carried out with the objective of selecting a small number of variables known to be associated with one or several mechanisms influencing sap flow. One of the most important process identified, freeze/thaw cycles, was assessed by testing several temperature variation thresholds between -3 and +5°C as well as mean daily temperature intervals to obtain the best predictors of the timing of sap flow (see S1 Table for the complete list of variables used and their description). For consistency, we maintain the term freeze/thaw cycle for climate variable names even when the threshold temperatures was not 0°C. Similarly, for cumulated degrees days above (thawing) and below (freezing), the threshold is always calculated relative to the threshold temperature considered. Since the freeze/thaw cycle and cumulative degree day calculations are inherently sensitive to any smoothing of the temperature signals that result from the averaging of multiple locations, weekly climate variables were first calculated for individual weather stations and subsequently averaged for each of the 11 regions.

**Table 1:** The number of producers surveyed within each region as well as the statistics on the starts, ends and lengths (Julian days) of the period of production.

Region	Number of producersSurveyed	Starts	Ends	Lengths
BSL	15	83±8 (67–98)	116±4 (109–121)	33±7 (21–42)
CTS	12	77±7 (66–91)	115±3 (109–119)	38±6 (28–49)
BEA	15	73±11 (60–98)	107±6 (95–118)	33±10 (14–49)
QUE	15	71±9 (60–91)	110±5 (95–115)	38±8 (21–49)
MAU	8	77±12 (60–98)	108±8 (88–117)	31±12 (8–35)
LAN	8	73±10 (54–91)	106±7 (94–115)	33±8 (21–56)
EST	12	73±11 (60–98)	108±6 (95–115)	35±10 (14–49)
LAU	10	73±13 (59–104)	110±10 (95–139)	37±9 (21–49)
CDQ	10	71±11 (54–91)	105±8 (93–115)	34±12 (21–56)
STY	8	66±9 (53–81)	102±5 (94–111)	34±10 (14–42)
VAL	8	65±7 (56–76)	101±6 (87–106)	34±7 (21–42)

Shown is the average± standard deviation as well as the range in brackets.

### Timing and duration of production season model

We modeled the duration of the production season, which was defined as the interval between the first (start) and the last (end) week of production for a given region and year. It is important to note that this definition may not always match exactly with the physiological sap flow period, but rather with the period during which maple sap is actively transformed into syrup. In some circumstances, the actual sap flow period may extend over the production season because the producers may cease to produce syrup because of low sap quality or volume, especially towards the end of the season. Our definition thus combines both the physiological and operational aspects of maple syrup production. Logistic regression was used to predict the duration, start and end of the production season (Eq 1). The outcome modelled in this case is whether production is achieved or not in a given week.

$$\hat{y}=\frac{e^{\beta_{1}x_{1}+{\beta_{2}x}_{2}+\ldots {\beta_{n}x}_{n})}}{1+e^{\beta_{1}x_{1}+{\beta_{2}x}_{2}+\ldots {\beta_{n}x}_{n})}}+\epsilon$$ (1)

Where $\hat{y}$ is the probability of a given week to have some production, β_1_ to β_n_ are the parameters to estimate, x_1_ to x_n_ the independent explanatory variables, and ε the residual error of the model.

A categorical variable was created to discriminate the weeks that belong to the period of production. To select the explanatory variables, Pearson’s correlation was calculated between the categorical variable and the climatic variables. Climatic variables with the largest correlations with the categorical variable were tested individually in a logistic model. The Akaike criterion (AIC) [17] was used to compare the different models. The model with the smallest AIC was selected and the correlation between the residuals of the selected model and the climatic variables was calculated. Variables with high correlation with the residuals were added individually to the initial model in order to produce improved models. Each improved model was compared to the initial model with a likelihood ratio test. If the likelihood ratio test detected a significant difference between the original model and the improved model, the improved model was retained. Among all the retained improved models, the model with the smallest value of AIC was kept and the precedent steps were repeated in an attempt to improve the model. These steps were repeated until additional variables did not improve the model. Once a final model was obtained, a ten-fold cross-validation was used to test model dependency on the "training" dataset used to calibrate it [18].

The cut-point (the value between 0 and 1 over which the week was considered as a production week) was first established at 0.5 and adjusted iteratively to 0.51 in order to maximise the sensitivity (ability of the model to accurately classify the production weeks) and specificity (ability of the model to accurately classify the non-production weeks) of the model.

### Climate scenarios

A set of 77 simulations from global climatic models (GCM) was used for syrup production season projections [19], belonging to three greenhouse gas (GHG) emission scenarios [20]. Simulated daily data, necessary to compute changes in sap flow dates and total production, were obtained for three temporal horizons: 1971–2000 (reference), 2046–2065 (2050 horizon) and 2081–2100 (2090 horizon). To build local climatic scenarios adapted for the projection of future maple syrup production, a post-processing method (“daily translation”) was applied on the raw climate model data variables of daily min and max temperatures and daily precipitation [21]. The required reference database of daily observations needed for the post-processing procedure was obtained from the ~10km x 10km gridded daily data described in Hopkinson et al. [22] and Hutchinson et al. [23].

### Projecting the production season timing

The model previously obtained for predicting the start, end and length of the production season was run with the 77 climatic scenarios to predict the impact of climate change on the timing and duration of the maple syrup production for each of the 11 regions (Fig 1). Weekly predictive climate variables used in the timing and duration were first calculated for all simulated years. For each of the 77 climate scenarios, each simulated year of data is used to determine the start, end and length of the syrup production season using the timing and duration model.

## Results and Discussion

### Variability in production period between regions and years

**Fig 2:**
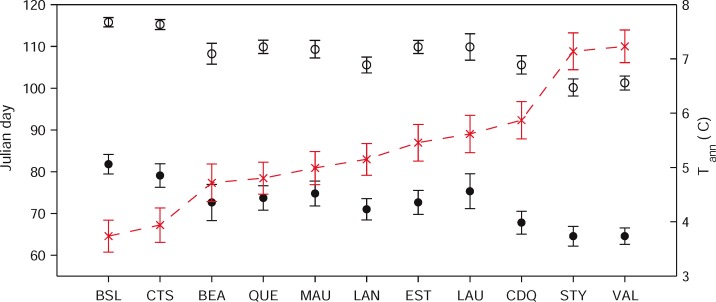
Variation in the start (closed circles) and end (open circles) of season (Julian days, left axis). Regions are ranked by increasing mean annual temperature (closed triangles, right axis).

**Fig 3:**
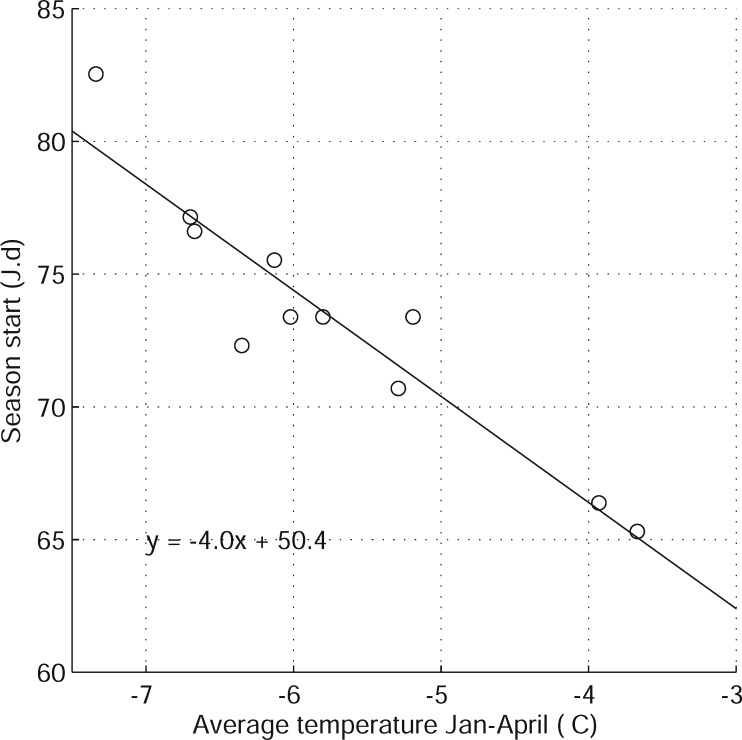
Relationship between average annual spring temperature and average Julian day of the beginning of the season of syrup maple production for the 11 regions studied. The equation is shown on the figure along with the r^2^.

The average day (Julian day) when the production of syrup started for the reference period ranged from 65 (VAL and STY) to 83 (BSL) between regions (Fig 2, Table 1). A similar range was also observed for the end of the production with average Julian days of 101 and 116 for the same regions, respectively. The average temperature for the period of January to April (T_J-A_), that ranged from -3.7 (VAL) to -7.3°C (BSL), was a strong predictor of the start and end date (not shown) for each region (Fig 3). Although such a relationship may appear likely, this is the first time the temperature sensitivity (corresponding to the slope of the relationship shown on Fig 3) of the “phenology” of maple syrup production is quantified. Based on the latter equation, the onset of syrup production is expected to advance by about four days for each degree of warming. This value is in good agreement with reported values of changes in the date of flowering and leafing for many plant species that fall within 2.5 to 5 days for a warming of 1°C [24]. Clearly, such a model based on average temperature cannot be used to predict the day at which the season will start for a given year but it can be used to predict the long term changes in the onset of the maple syrup production using only scenarios of annual temperature (see below).

Despite the fact that the period of observations is not long in terms of absolute number of years (n = 13 years), the data set included strong between years and between regions variability in terms of weather and timing of maple syrup production. Weekly data for the years 2010 and 2008, the years with the earliest and the latest production periods, respectively, showed a 4–5 week difference in the timing of peak syrup production (data not shown). On a regional and yearly basis, the earliest date of onset of syrup production, Julian day 53, was observed in the STY region in 2010 while the latest (Julian day 104) was observed in LAU in 2003 (Table 1). Ends of the production period showed similar variability with the earliest and latest ends of production being observed on Julian day 87 (VAL, 2000) and 139 (LAU, 2003), respectively. Given that when the production starts later it normally ends later, the average length of the production season was much less variable than start and end dates (Fig 2; Table 1), ranging from 31 (MAU) to 38 Julian days (CTS and QUE).

### Modelling the season of maple syrup production with the logistic model

For explaining/predicting the production or absence of production of syrup in a given week, the following model was obtained:

$$\mathrm{Production}=\text{-}\;5.09+0.722\;\mathrm{FrThw}3:\mathrm{NbCum}–0.014\;{\left(\mathrm{FrThw}3:\mathrm{NbCum}\right)}^{2}–0.07\;\mathrm{GDDCum}$$ (2)

The logistic model is made of the cumulated number of freeze/thaw events with a threshold of 3°C (FrThw3:NbCum) along with the same variable squared. It has often been mentioned that freeze-thaw cycles (with a 0°C threshold) are necessary to induce sap flow for sugar maple. The threshold of 3°C obtained here may indicate that fluctuations around a given temperature close to 0°C are sufficient for sap flow to occur. However, the 3°C threshold is the one measured at the meteorological stations disseminated within each region. It is possible that the sugar maple stands under study may be located at higher elevation and that they were exposed to temperature variations closer to 0°C than 3°C. The model also includes the cumulated number of growing degree-days starting on Julian Day 1 using a 5°C threshold (GDDCum). This latter variable has a negative effect on the number of weeks with maple syrup production. Its role in the model is likely for predicting the end of the production period when too much heat has been accumulated by the maple stands. Overall, the model was successful at classifying weeks in the proper bin (production or not). The global model accurately predicted 83% of the production weeks and 95% of the non-production weeks (data not shown).

### Projections of future changes in the timing of the production season

The logistic model (Eq 2) was used with the climatic data of 77 scenarios to project future dates of start and end of the season of syrup production, as well as its length. The absolute values as well as the delta (changes as compared to the reference period 1971–2000) are shown in Table 2. Depending on the regions, the logistic model predicts that the start and end of the production season will occur 9–13 days, and 15–19 days earlier for the 2046–2065 and 2081–2100 periods respectively, as compared to the reference period (Table 2). Given that changes in season start and end of production dates are nearly similar, the simulations predict no changes in season length for the 2046–2065 period and only a small (1–2 days) but insignificant increase for the 2081–2100 period. Overall the magnitude of the changes predicted here are coherent with the results of Skinner et al. [4] who predicted shifts in the sap season in the order of 15 and 30 days for 2100 using only the B1 and A1fi climate change scenarios, respectively. The B1 scenario is more similar to the median scenario used in our simulations while the A1fi scenario is one of the most extreme in terms of increased temperature which explains the particularly strong changes (30 days) in season timing obtained with the latter scenario.

**Table 2:** Projections (average ± standard deviation) for the start, end (Julian days; jd), and length (days; d) of production season and the total annual production for the reference period 1971–2000, and two future periods, 2046–2065 and 2081–2100.

		Mean average date for each period				Mean average change relative to 1971–2000	
		(n = 77 scénarios)				(n = 77 scénarios)	
Region	Variable	(1971–2000)	(2046–2065)	(2081–2100)		(2046–2065)	(2081–2100)
**BSL**	Start (J.d)	86.2 ± 1.4	76.5 ± 5.3	69.3 ± 7.9		-9.7 ± 5.0	-16.9 ± 7.7
	End (J.d)	123.1 ± 0.8	114.0 ± 5.4	108.2 ± 7.7		-9.1 ± 5.2	-14.8 ± 7.5
	Length (d)	43.9 ± 1.9	44.5 ± 6.3	45.6 ± 8.1		0.6 ± 5.8	1.7 ± 7.5
**CTS**	Start (J.d)	81.3 ± 1.5	71.1 ± 5.1	64.3 ± 7.0		-10.3 ± 4.8	-17.0 ± 6.7
	End (J.d)	118.3 ± 0.9	108.6 ± 4.7	102.8 ± 7.1		-9.7 ± 4.5	-15.5 ± 6.9
	Length (d)	44.0 ± 2.0	44.2 ± 6.3	44.9 ± 8.7		0.2 ± 5.3	0.9 ± 7.5
**BEA**	Start (J.d)	74.5 ± 1.9	62.7 ± 5.3	55.5 ± 7.1		-11.8 ± 5.5	-19.0 ± 7.2
	End (J.d)	114.1 ± 1.1	103.2 ± 5.1	97.2 ± 7.1		-10.9 ± 4.8	-17.0 ± 6.9
	Length (d)	46.6 ± 2.5	47.1 ± 7.6	47.8 ± 9.2		0.5 ± 7.0	1.2 ± 8.2
**QUE**	Start (J.d)	82.5 ± 1.8	72.4 ± 4.9	65.9 ± 6.6		-10.0 ± 4.5	-16.6 ± 6.4
	End (J.d)	118.2 ± 0.8	108.7 ± 4.6	103.3 ± 6.6		-9.5 ± 4.5	-14.9 ± 6.4
	Length (d)	42.7 ± 2.0	42.9 ± 6.2	43.8 ± 8.1		0.3 ± 5.2	1.1 ± 7.0
**MAU**	Start (J.d)	82.5 ± 1.6	73.5 ± 4.7	67.3 ± 6.3		-9.0 ± 4.2	-15.1 ± 5.9
	End (J.d)	116.7 ± 0.9	107.8 ± 4.7	102.7 ± 6.1		-8.9 ± 4.6	-14.0 ± 5.9
	Length (d)	41.2 ± 1.9	41.0 ± 6.2	41.8 ± 7.4		-0.3 ± 5.3	0.6 ± 6.3
**LAN**	Start (J.d)	77.3 ± 1.5	67.0 ± 4.8	60.4 ± 6.6		-10.4 ± 4.6	-16.9 ± 6.2
	End (J.d)	113.5 ± 0.8	103.2 ± 4.9	98.0 ± 6.3		-10.3 ± 4.7	-15.5 ± 6.1
	Length (d)	43.1 ± 1.9	43.0 ± 6.6	44.1 ± 7.9		-0.2 ± 5.9	1.0 ± 6.9
**EST**	Start (J.d)	67.5 ± 1.9	55.0 ± 5.7	49.0 ± 7.7		-12.4 ± 6.1	-18.4 ± 7.6
	End (J.d)	109.4 ± 1.1	98.2 ± 5.7	91.7 ± 7.6		-11.2 ± 5.3	-17.6 ± 7.2
	Length (d)	48.9 ± 2.3	49.6 ± 8.5	48.8 ± 10.4		0.7 ± 8.2	-0.1 ± 9.6
**LAU**	Start (J.d)	76.3 ± 1.5	64.7 ± 5.5	57.9 ± 6.8		-11.6 ± 5.5	-18.4 ± 6.6
	End (J.d)	111.6 ± 0.9	101.3 ± 4.9	95.8 ± 6.7		-10.3 ± 4.7	-15.8 ± 6.6
	Length (d)	42.3 ± 1.7	43.3 ± 7.0	44.6 ± 7.7		1.0 ± 6.6	2.3 ± 7.1
**CDQ**	Start (J.d)	69.4 ± 1.7	56.3 ± 5.3	50.1 ± 7.2		-13.1 ± 5.6	-19.3 ± 7.1
	End (J.d)	108.7 ± 1.1	98.4 ± 4.6	92.3 ± 6.9		-10.3 ± 4.4	-16.4 ± 6.7
	Length (d)	46.2 ± 2.2	48.7 ± 7.2	48.6 ± 9.1		2.4 ± 6.9	2.4 ± 8.5
**STY**	Start (J.d)	61.7 ± 1.6	48.3 ± 6.3	42.9 ± 7.6		-13.4 ± 6.4	-18.8 ± 7.5
	End (J.d)	103.5 ± 1.4	92.7 ± 5.0	86.2 ± 7.0		-10.8 ± 4.7	-17.3 ± 6.8
	Length (d)	48.8 ± 1.9	50.8 ± 8.5	49.7 ± 10.0		2.1 ± 7.9	0.9 ± 9.3
**VAL**	Start (J.d)	60.6 ± 1.6	47.2 ± 6.3	42.1 ± 7.5		-13.3 ± 5.9	-18.5 ± 7.0
	End (J.d)	102.7 ± 1.3	91.7 ± 4.9	85.9 ± 6.9		-11.0 ± 4.6	-16.8 ± 6.8
	Length (d)	49.1 ± 2.3	51.0 ± 8.5	50.0 ± 10.1		1.9 ± 7.5	0.9 ± 8.9

Shifts in season start and end between the reference (1971–2000) and future periods are also reported as delta days; d). It is important to note that the ± standard deviation values reported are indicative of uncertainty in the mean Julian date between the 77 climate scenarios and do not represent year to year variability. Please see Fig 1 title for region acronyms.

The projections obtained with the logistic model were compared with the projections of the model based on average T_J-A_ temperature. For this comparison, the emphasis was put on the start of the production season since the length is not projected to be affected in the future. Moreover, the start of the season is crucial from an operational point of view for the producers since they normally tap the maple trees a few weeks before they expect the season to begin. On some occasions, if sap flow starts much earlier than usual, the producers may not be ready to collect sap and may face economic losses.

**Fig 4:**
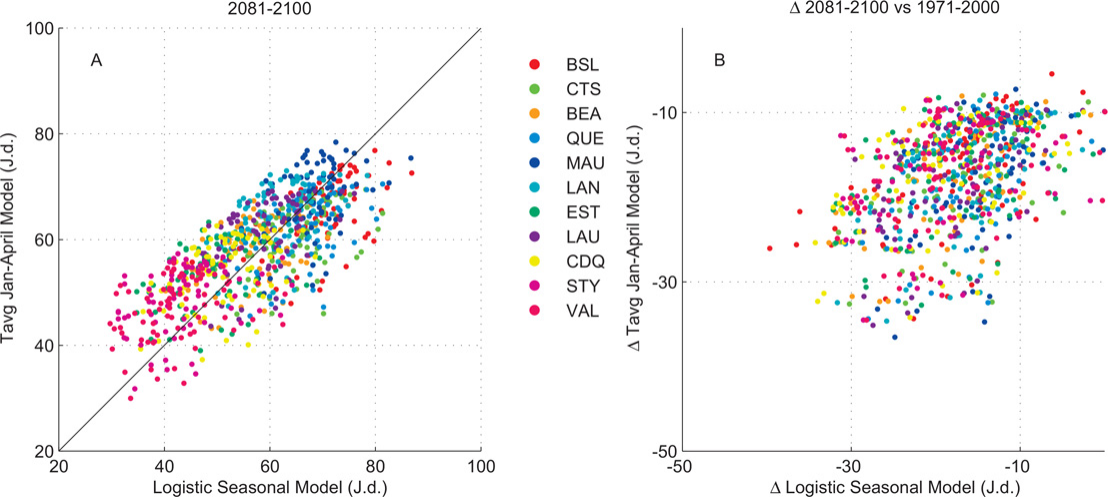
Relationship between the Julian day of the start of the season production (left panel, A) as projected with the logistic model (x axis) and the T_J-A_ model (y axis) for the 2081–2100 period for each of the 77 simulation for the 11 regions (n = 847). The right panel (B) shows the distribution of the displacement (delta Julian day) of the 2081–2100 vs the 1971–2000 period.

The comparison between the projections of the two models is shown in Fig 4 for the first day of production (panel A) as well as for its displacement in time (panel B) for the 2081–2100 period (n = 847, 77 simulations x 11 regions). There was a strong agreement between the two models (r^2^ = 0.80, p < 0.001) regarding the first day of production which was mainly driven by the regional gradient (both models are ranking the regions fairly in the same order since there is an average annual temperature gradient between regions, Fig 2). The displacement in time of the first day of production (panel B) was also comparable in terms of dispersion (similar variability and range) and both models yielded identical average displacements of -17.5 ± 7 days (n = 847). A strong correlation between the numbers of days of displacement was not expected (as compared to day of start, panel A) since there is no major regional differences in the projection of displacement (Table 2). The large number of simulations as well as the very good agreement of the two models which are based on different and weakly interrelated variables, suggest a robust assessment of the projected changes in the future. The results mean that sugar maple trees, as well as syrup producers, must adapt to seasons that will start earlier than at present which appears plausible given that past observations included some very early and very late seasons.

**Fig 5:**
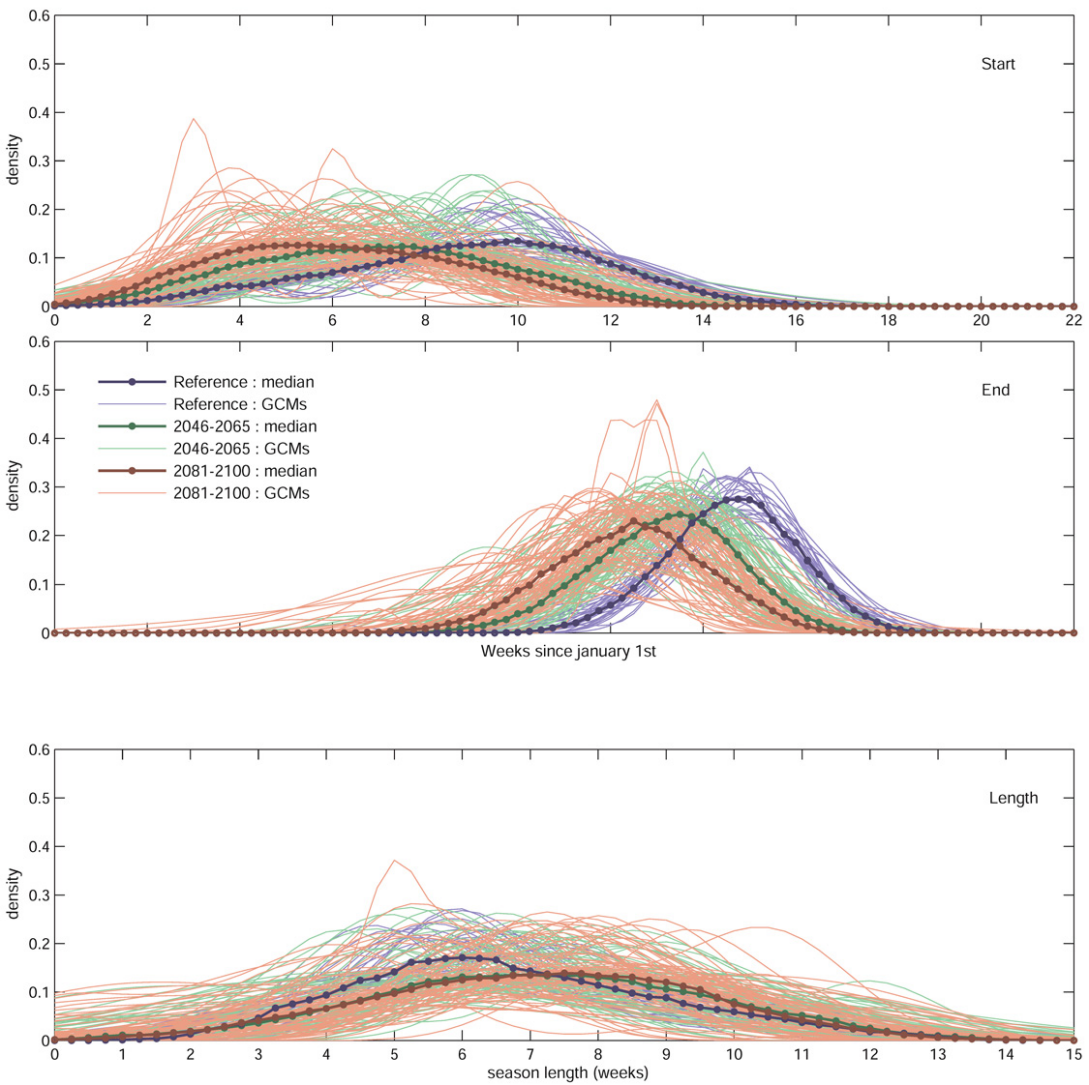
Probability density for the start (A), end (B) and length (C) of production season for the Saint-Jean Valleyfield region for the 1971–2000, 2046–2065 and 2081–2100 periods, respectively. Each thin line represents the distribution of the different years within each of the 77 projections based on the 77 global climate scenarios.

In our study, the use of a great number of climatic scenarios is useful not only for obtaining a robust estimation of changes in the timing of the maple syrup production season, but also to assess the variability around the average estimates. Although the use of the median result of many climatic simulations is seen as robust [15], there is quite a lot of variation among the 77 simulations for a given region. An example of the detailed results obtained with the logistic model is illustrated on Fig 5 for the Valleyfield region (VAL). The density probability curves of each of the date of onset (panel A) or ends (panel B) of production for a given year obtained by each of the 77 individual simulations and for each period of time considered are shown as well as for season lengths (panel C). Overall, our projections for the start, end and length of the production season tend to suggest an increase in variability for the future, as shown by the density probability curves that show an increasing spread (panels B and C for date of end and season lengths) in future projections and also by the increasing standard deviation coefficient in the period 2041–2060 and 2081–2100 as compared to the 1971–2000 period (Table 2). However, this apparent increase could be simply due to the large number of climate scenarios used (based on multiple climate models, driven with different scenarios of GHG emissions), whereas only one "scenario" will be realized in the future. In fact, total climate projection uncertainty is known to increase into the future as families of GHG emissions (SRES families) diverge and by the fact that the various climate models have different sensitivities to a given amount of GHG forcing [25]. To investigate the potential for a higher variability in the future, we computed the standard deviation in annual production for each period (n = 20 years each) and simulation. In other words, we compare the variation in the results obtained for each simulation between periods, not among simulations. We could not detect a significant change in variation for the future (Table 3). We thus concluded that the shift to an earlier sap season in the future will not be accompanied by an increase in between year variability in the timing of maple syrup production.

**Table 3:** Year to year variability (mean standard deviation ± 1 SD) for the start and end, and duration of the season for the three study periods.

		standard deviation for each period		
		(n = 77 scenarios)		
Region	Variable	(1971–2000)	(2046–2065)	(2081–2100)
**BSL**	Start (J.d)	12.3 ± 2.4	13.2 ± 2.9	14.4 ± 3.5
	End (J.d)	7.4 ± 1.6	7.7 ± 1.9	8.8 ± 2.6
	Length (d)	9.6 ± 1.9	11.2 ± 2.9	12.4 ± 3.3
**CTS**	Start (J.d)	14.3 ± 2.6	14.9 ± 3.1	15.5 ± 3.4
	End (J.d)	7.8 ± 1.8	8.4 ± 2.0	9.5 ± 2.6
	Length (d)	11.3 ± 2.0	13.4 ± 3.2	13.8 ± 3.7
**BEA**	Start (J.d)	16.4 ± 2.0	16.4 ± 3.1	16.8 ± 3.0
	End (J.d)	8.5 ± 1.6	9.4 ± 2.4	10.7 ± 2.9
	Length (d)	13.4 ± 2.2	15.2 ± 2.9	15.6 ± 3.7
**QUE**	Start (J.d)	12.6 ± 2.5	13.2 ± 2.9	14.4 ± 3.3
	End (J.d)	7.4 ± 1.6	8.1 ± 2.0	9.0 ± 2.5
	Length (d)	9.9 ± 2.2	12.0 ± 3.1	12.9 ± 3.3
**MAU**	Start (J.d)	12.0 ± 1.9	12.8 ± 2.9	14.0 ± 3.5
	End (J.d)	7.6 ± 1.5	8.4 ± 1.9	8.9 ± 2.1
	Length (d)	9.6 ± 1.7	11.6 ± 2.8	12.4 ± 3.3
**LAN**	Start (J.d)	13.9 ± 2.0	14.7 ± 3.0	15.3 ± 3.5
	End (J.d)	8.1 ± 1.4	8.9 ± 2.1	9.5 ± 2.4
	Length (d)	11.1 ± 2.2	13.2 ± 2.8	13.3 ± 3.3
**EST**	Start (J.d)	17.7 ± 2.4	17.0 ± 2.9	16.2 ± 2.6
	End (J.d)	9.3 ± 2.1	9.9 ± 2.4	11.1 ± 2.3
	Length (d)	14.9 ± 2.3	15.7 ± 2.8	15.3 ± 3.2
**LAU**	Start (J.d)	14.0 ± 1.9	15.4 ± 3.0	16.1 ± 2.9
	End (J.d)	8.2 ± 1.6	9.0 ± 2.1	9.7 ± 2.3
	Length (d)	11.6 ± 2.0	14.0 ± 2.9	13.8 ± 3.1
**CDQ**	Start (J.d)	16.8 ± 2.0	16.8 ± 3.0	16.6 ± 2.7
	End (J.d)	8.2 ± 1.5	8.9 ± 1.9	10.3 ± 3.0
	Length (d)	13.8 ± 2.0	15.5 ± 3.1	15.2 ± 3.5
**STY**	Start (J.d)	17.8 ± 1.7	16.5 ± 3.0	15.5 ± 3.0
	End (J.d)	8.9 ± 1.9	9.6 ± 2.2	10.8 ± 2.5
	Length (d)	15.0 ± 2.0	15.7 ± 2.8	14.9 ± 3.3
**VAL**	Start (J.d)	18.6 ± 1.9	16.7 ± 2.9	15.6 ± 2.9
	End (J.d)	8.9 ± 1.7	9.8 ± 2.3	10.6 ± 2.5
	Length (d)	15.7 ± 1.8	15.8 ± 2.7	15.1 ± 3.4

The standard deviation reported is indicative of uncertainty between the 77 climate scenarios. Please see Fig 1 for region acronyms.

The data for the VAL region also show that a few simulations for the 2081–2100 period are projecting years with short seasons (1–3 weeks), or even no season at all (Fig 5C; red lines crossing the Y axis—intercept = 0 means no season). These rare events will correspond to “extreme” years in a climate where the average start of the season for the VAL region will be displaced by 19 days (Table 2). These events will be more frequent for the southernmost regions (data not shown), where the climate is already warmer but since their absolute frequency is still projected to be small, no significant impacts on between year variability are projected. Interestingly, such effect (quasi-absence of a season resulting in a low syrup yield) was already observed in several US states [26] in the exceptionally warm spring of 2012, perhaps telling of what the future holds for these regions.

## Conclusion

In this study, we built models capable of predicting the start and the end (and thereby length) of the maple syrup production period based on a unique data set of weekly observations made at 121 maple stands spread in 11 regions of the province of Québec (which is responsible for 75% of the world sugar maple syrup production). There was a strong spatial and temporal variability in the timing of the production period. In warmer regions, production began on average 17 days earlier as compared to colder regions. The average date of the beginning of production can be predicted with success with the average temperature from January to April. A logistic model using climatic variables associated with the physiological process of sap flow was also built from the 143 individual observations (13 years x 11 regions) for predicting the period of syrup production. When used in combination with 77 global climate scenarios, the models predict that the period of production will begin 9–13 and 15–19 days earlier for the 2046–2065 and 2081–2100 periods, respectively, but its length is not projected to change. The strong agreement between the two independent models suggests a robust assessment of the changes in timing. The results also show that there will be a higher frequency of extreme years with short season of production in the future, especially in the southern part of the province. However, according to our projections, this would not be sufficient to quantitatively increase the inter-annual variability in the dates of beginning and end of the production season. Given that predicting “extreme” years with very short season of production is more difficult than predicting “average years” in terms of production season timing, and considering that the data available for model calibration covered a relatively short period of time (13 years), caution is warranted when interpreting the projected occurrence of “short seasons” in the future. More research is needed to further investigate this possibility and to assess the potential impacts on maple syrup yield.

Overall, the results show that syrup producers will be facing sap seasons starting earlier than at present which means that they must adapt by being ready to tap the sugar maple trees earlier in the season. On the other hand, the projected stability of the inter-annual variability in the beginning of the sap season, indicate that the syrup producers will not be confronted to a greater between years uncertainty with regards to the moment of tapping the trees.

## Supporting Information

S1 Table Description of climate variables used in the construction of statistical models. (DOCX)
