# Supplementary material for: Impacts of Climate Change on the Timing of the Production Season of Maple Syrup in Eastern Canada
Source: PLoS One. 2015 Dec 18;10(12):e0144844. doi: 10.1371/journal.pone.0144844 (PMC4684277; doi:10.1371/journal.pone.0144844)
Supplement: S1 Table — (DOCX) [file pone.0144844.s001.docx]

S1 Table. Description of climate variables used in the construction of statistical models.

| **Variables** | **Description** |
| --- | --- |
| FrzThwX :Nb | Weekly number of freeze / thaw events with a ‘freeze’ threshold equal to the value of X (varies between -3 and 5°C). |
| FrzThwX :NbCum | Weekly cumulated sum of FrzThwX :Nb beginning January 1^st^. |
| FrzThwX :CumFrz | Weekly sum of degrees below X°C during freeze /thaw events of threshold X (from -3 to 5°C). Weather stations with no freeze / thaw events for a given week are given a value of 0. |
| FrzThwX :CumThw | Weekly sum of the degrees above X°C during freeze /thaw events of threshold X (from -3 to 5°C). Weather stations with no freeze / thaw events for a given week are given a value of 0. |
| FrzThwX :IntensFrz | Weekly average freeze intensity of freeze / thaw events of threshold X°C. Calculated as FrzThwX :CumFrz/ FrzThwX :Nb |
| FrzThwX :IntensThw | Weekly average thaw intensity of freeze / thaw events of threshold X°C. Calculated as FrzThwX :CumThw/ FrzThwX :Nb |
| FrzThwX :Trng | Temperature range between daily maximum and minimum temperatures during freeze thaw events of threshold X°C. |
| GDD | Weekly sum of growing degree days >=5°C (Days with average temperature of < 5°C are given a value of 0) |
| GDDCum | Weekly cumulated sum of GDD beginning January 1^st^. |
| Pr | Weekly total precipitation |
| Pr_Janv | Total precipitation for January |
| TavgJanv | Average daily temperature for January |
| Tavg0_8C :Nb | Weekly number of days with daily average temperatures between values of 0 and 8°C. |
| Tavg0_8C :NbCum | Weekly cumulated sum of Tavg0_8C :Nb since January 1^st^ . |
| Tavg0_6C :Nb | Weekly number of days with daily average temperatures between values of 0 and 6°C. |
| Tavg0_6C :NbCum | Weekly cumulated sum of Tavg0_6C :Nb since January 1^st^ . |
| TmaxJanv | Maximum weekly average of daily maximum temperatures for the month of January |
| TminJanv | Minimum weekly average of daily minimum temperatures for the month of January |
| WTavg | Weekly average temperature |
| WTavgMin | Weekly average of daily minimum temperatures |
| WTavgMax | Weekly average of daily maximum temperatures |
